# Neuro-computational foundations of moral preferences

**DOI:** 10.1093/scan/nsab100

**Published:** 2021-09-06

**Authors:** Giuseppe Ugazio, Marcus Grueschow, Rafael Polania, Claus Lamm, Philippe Tobler, Christian Ruff

**Affiliations:** Zurich Center for Neuroeconomics (ZNE), Department of Economics, University of Zurich, Zurich 8006, Switzerland; Moral Psychology Research Lab, Department of Psychology, Harvard University Cambridge, Cambridge, MA, USA; Geneva Finance Research Institute, University of Geneva, Geneva, Switzerland; Zurich Center for Neuroeconomics (ZNE), Department of Economics, University of Zurich, Zurich 8006, Switzerland; Decision Neuroscience Lab, Department of Health Sciences and Technology, ETH Zurich, Zurich, Switzerland; Social, Cognitive and Affective Neuroscience Unit, Department of Basic Psychological Research and Research Methods, University of Vienna, Vienna, Austria; Zurich Center for Neuroeconomics (ZNE), Department of Economics, University of Zurich, Zurich 8006, Switzerland; Zurich Center for Neuroeconomics (ZNE), Department of Economics, University of Zurich, Zurich 8006, Switzerland

**Keywords:** moral preferences, subjective value, common currency neural network

## Abstract

Moral preferences pervade many aspects of our lives, dictating how we ought to behave, whom we can marry and even what we eat. Despite their relevance, one fundamental question remains unanswered: where do individual moral preferences come from? It is often thought that all types of preferences reflect properties of domain-general neural decision mechanisms that employ a common ‘neural currency’ to value choice options in many different contexts. This view, however, appears at odds with the observation that many humans consider it intuitively wrong to employ the same scale to compare moral value (e.g. of a human life) with material value (e.g. of money). In this paper, we directly test if moral subjective values are represented by similar neural processes as financial subjective values. In a study combining functional magnetic resonance imaging with a novel behavioral paradigm, we identify neural representations of the subjective values of human lives or financial payoffs by means of structurally identical computational models. Correlating isomorphic model variables from both domains with brain activity reveals specific patterns of neural activity that selectively represent values in the moral (right temporo-parietal junction) or financial (ventral-medial prefrontal cortex) domain. Intriguingly, our findings show that human lives and money are valued in (at least partially) distinct neural currencies, supporting theoretical proposals that human moral behavior is guided by processes that are distinct from those underlying behavior driven by personal material benefit.

Moral preferences play a crucial role in determining how we perceive the world, how we act and what we like. Differences in moral preferences lie at the heart of many types of conflicts between individuals and groups (Cohen *et al.*, 2006; Koleva *et al.*, 2012; Goodwin *et al.*, 2014) and have even led to wars between nations (Berns and Atran, 2012; Iyengar and Westwood, 2015; Kovacheff *et al.*, 2018). More generally, such differences in moral preferences account for the substantial variation in how we judge the actions of other humans and artificial agents (Awad *et al.*, 2018). Given the relevance and timeliness of moral preferences, it is remarkable how little we understand about the neural and cognitive mechanisms that determine moral decision-making. Knowledge about these processes is essential for understanding cultural and individual differences in moral perception and behavior (Graham *et al.*, 2009; Haidt, 2012; Greene, 2015) and for the development of artificial intelligence that concurs with the human understanding of morality (Malle, 2016).

In choice domains other than morality, such as financial decisions, individual preferences have been studied intensely in terms of neural processes that assign values to choice options (Schultz, 2006). These values are usually inferred by observing choices and fitting models of the presumed utility derived from characteristics of the choice options (such as their magnitude and price). Note that this assigned utility differs between individuals and, therefore, is not identical with representations of the option characteristics themselves (since people differ in how they value these characteristics). While most economic models do not actually assume that utilities are represented cardinally, neuroscientists have nevertheless shown that presumed value signals derived with such models correlate with brain activity (Clithero and Rangel, 2014). For instance, several studies have demonstrated that a person’s economic preferences are reflected in subjective values encoded by the activity of the ventral-medial prefrontal cortex (vmPFC), ventral striatum (VS) and posterior cingulate cortex (PCC) (Kable and Glimcher, 2007; Hare *et al.*, 2008; Clithero and Rangel, 2014; Grueschow *et al.*, 2015). Based on these findings, it has been proposed that the brain values choice options on a common scale that may allow us to compare and choose efficiently across many different types of goods. This is thought to hold not only for material goods (e.g. art, food or money) but also for non-material values [e.g. beauty, praise, or status (Izuma *et al.*, 2008; Zink *et al.*, 2008; Levy and Glimcher, 2012)].

In the domain of moral decision-making, several studies (Hsu *et al.*, 2008; Shenhav and Greene, 2010; Hutcherson *et al.*, 2015a; Crockett *et al.*, 2017) have likewise proposed that computing the value of human lives or of human pain may draw on the same neural mechanisms that are involved in computing values of non-moral goods. For instance, the vmPFC has been reported to represent the ‘expected values’ of moral options (i.e. the number of possible deaths multiplied by their respective probability of occurrence) (Shenhav and Greene, 2010). However, as this expected-value computation is objective and, therefore, identical across different agents, it does not account for a given individual´s ‘subjective valuation’ of the different options that underly choices between them. Another study showed differences in the neural correlates of emotional and utilitarian appraisals during moral decisions (Hutcherson *et al.*, 2015a); during utilitarian appraisals, the vmPFC represented the number of people to be saved. Similarly, an earlier study (Hsu, 2010) reported that the caudate is involved in representing the utility of the outcomes of a moral choice. These findings, however, reveal neither if differences in moral preferences result from different sensitivities to these attributes nor if individuals who assigned different weights to these attributes would take different moral decisions. Furthermore, recent studies (Tricomi *et al.*, 2010; Hare *et al.*, 2010; Crockett *et al.*, 2014, 2017) showed that neural value responses in the vmPFC were differentially modulated during choices about financial rewards that were coupled with morally relevant consequences (e.g. painful shocks to either others or oneself). However, in this context, it is impossible to know whether these neural activations indeed reflect moral concerns rather than other aspects of these consequences. For instance, in the case of the pain studies (Crockett *et al.*, 2014, 2017), the vmPFC could be responding to differences in the representation of others’ *vs* one’s own affective states during pain (Lamm *et al.*, 2007; Silani *et al.*, 2013). Moreover, since these decisions always entailed trade-offs between pain and monetary profit, the observed neural responses in the value system reflected the valuation of material goods (and how this was altered by different moral contexts). Finally, previous studies of prefrontal cortex (PFC) lesion or psychiatric patients observed altered moral judgments and behaviors (Greene, 2007; Zucchelli and Ugazio, 2019); however, none of these studies investigated whether these changes in moral judgments reflect changes in moral value computations or other more general impairments, for instance, the reduced ability to integrate emotional feedback (especially negative) into action planning (Blair, 2007a,b). Thus, despite several interesting findings on moral decision-making and value computations, we still do not know the neural origins of an individual’s subjective moral preferences and whether the neural mechanisms implementing purely moral value computations differ from those involved in the neural valuation of material goods. Clarifying whether moral and material preferences are represented by distinct neural mechanisms is essential for a better understanding of decision-making processes that entail a combination of these two types of preferences, such as philanthropy, sustainable finance or political decisions trading-off economic outcomes with citizens’ health risks.

Differences in the neural processes underlying moral and material preferences are suggested by theoretical accounts emphasizing that moral preferences may originate from specific value-computation mechanisms. These accounts rest on the observation that many people perceive human lives as having an intrinsic (sacred) value (Sandel, 2012; Dogan *et al.*, 2016) that cannot, and should not, be measured on the same scale as the value of material objects (Kleinig, 1991). For example, widespread outrage is usually observed when people realize that the value of human lives is monetarily quantified, for instance, during choices between health policies (Kmietowicz, 2001) when determining how to prioritize admissions to overloaded intensive care units (Palladino *et al.*, 2020), in the context of a company’s decision on whether to recall a dangerous car model (Dowie, 1977) or when people are traded for money (Chuang, 2006). Based on these observations, it has been proposed that assigning a financial value to a human life appears intuitively wrong for many people (Sandel, 2012). This suggests that moral valuation may be implemented by processes that are distinct from those involved in the valuation of material goods. Importantly, in order to dissociate moral preferences (and the underlying neurocognitive processes) from other types of preferences, such as conventional, financial or social preferences, it is necessary to clearly define what makes a preference ‘moral’. In line with established definitions, we consider moral preferences to be those social preferences that are universalizable (i.e. that apply categorically to all situations and individuals), independent from the presence of a given authority and concerned with justice, harm or other rights (Eggleston and Turiel, 1985; Kelly *et al.*, 2007; O’Neill, 2017; Stich, 2018). According to this definition, moral preferences are clearly distinguishable from social and conventional preferences. For example, the moral preference to not harm others is clearly distinguished by these criteria from simple social preferences, such as how to share resources among friends, and conventional preferences, such as placing table silverware in a given order. This has been demonstrated by a large body of literature showing that already from a young age, humans distinguish between situations regulated by moral *vs* social/conventional preferences (Smetana, 1981; Blair, 1996; Nucci, 2006; Prinz, 2006; Smetana *et al.*, 2014). Previous research also suggests that there may be neural networks specifically dedicated to processing moral information (Moll *et al.*, 2008), which may or may not overlap with a valuation network responsible for processing social information in general (Ruff and Fehr, 2014). Note that this view does not imply that moral/sacred and values cannot be compared at all, only that they may be processed by different systems before they can be compared (in a presumably non-habitual manner) whenever this may be required.

In the present work, we test this alternative hypothesis by identifying where and how the brain computes purely subjective moral values and by explicitly comparing the neural instantiation of moral and financial value computations. We measured these two types of subjective valuation processes with structurally equivalent choice tasks that differed only in the content of the choice options: valuing human lives for moral decisions and valuing monetary rewards for financial decisions. We decided to focus on human lives since subjective moral values are essential for the difficult decisions whether some lives are more valuable than others and since there are considerable individual differences in this regard (Awad *et al.*, 2018). One example is decisions about recipients of an organ transplant, for which it is often required to implement a policy ranking among the potential recipients to decide who is most deserving of receiving the organ (Courtney and Maxwell, 2009). We adapted this decision situation to study the neural representations of subjective moral values, which we derived by fitting standard computational models of value-based decision-making to the observed choices (Chung and Herrnstein, 1967; Rubinstein, 2003; Green *et al.*, 2004) and by correlating the estimated subjective values with neural activity as measured with functional magnetic resonance imaging. More specifically, we used the standard econometric ‘revealed-preference’ approach to estimate the value that each participant assigns to not sacrificing a given person (who is characterized by morally relevant previous deeds; see Materials and Methods below for more details) in order to save a varying, larger group of other people. We did so by applying a standard value-discounting choice model to the individually observed choices, which resulted in a trial-wise subjective value measure. Note that this subjective value will vary systematically across trials, but also across participants, in line with their moral preference (as derived from the fitted choice model). That is, an individual with a strong moral preference for protecting individual lives will consider it immoral to harm someone even if this can bring about a greater good and will assign a very high subjective value to the life of the person that may be harmed. Conversely, an individual with a strong moral preference for bringing about the greater good will assign a low subjective value to the life of the person that will be harmed in order to achieve such greater good. Thus, a given trial/choice problem will elicit very different subjective values (SVs) in participants with different preferences (between-subject variation), and the same participant will assign very different SVs to different choice problems/trials based on the varying moral deservingness and number of people that can be saved on this trial (within-subject variation). By correlating these varying subjective values with BOLD signals within subjects, we can thus identify neural responses that reflect individually specific value computations that are fully in line with each individual’s moral preference rather than with the objective magnitudes/probabilities/delays characterizing a choice problem [which would not vary across individuals with different preferences, as in e.g. Shenhav and Greene (2010) and Hutcherson *et al.*  (2015b)].

In order to fully capture individual behavioral variability during both decision types, we varied the decision-relevant characteristics of the choice options along two dimensions. For the financial decisions, participants chose between options that differed in terms of both the monetary amount and the temporal delay at which the amounts would be paid out. The subjective value of the choice options, therefore, depends inherently on individual time preferences (Green and Myerson, 2004; McClure, 2004; Kable and Glimcher, 2007), which determine how the reward magnitude (i.e. the amount of money one can receive) is discounted by the delay (i.e. the number of days) one has to wait until receiving the reward. The moral decisions were constructed to match exactly this structure: They consisted of a customized moral scenario similar to the classic trolley moral dilemma (Foot, 1967) that required participants to take medical decisions similar to real-life moral choices taken by doctors: participants had to choose between (i) interrupting a coma patient’s life support to use the patient’s organs to save the lives of other individuals or (ii) leave the coma patient on life support and let the other individuals die (see Materials and Methods below). For these moral decisions, we parametrically varied both the choice-relevant magnitude (i.e. the number of lives one could save) and a second factor that discounted the value of the lives at stake. This factor was the moral deservingness of the person that would have to be sacrificed in order to save the others (as indicated by different prior criminal records of this person). Both these factors have been shown to play important roles in moral judgments (Kliemann *et al.*, 2008; Shenhav and Greene, 2010). Interestingly, there seem to be fundamental cultural and individual differences in the importance assigned to moral deservingness when people have to estimate and compare the value of human lives (Awad *et al.*, 2018). This highlights the importance of understanding how individual and situational factors jointly determine moral perception and preferences. Our paper takes an important step in this direction since it provides a value-computation model that captures moral preferences both behaviorally and in terms of the underlying neural value computations.

Our setup allowed us to directly compare the neural value representations underlying both types of choices in the same participants using functional magnetic resonance imaging (fMRI). We ensured that the perceptual and sensorimotor demands required by both types of choices were kept similar, as the choice screens in both contexts were arranged analogously (Figure 1A and B) and as responses were given with the same motor actions. Based on the existing value-based literature, we estimated subjective values underlying the financial choices by means of computational modeling (Frederick, 2003; Rubinstein, 2003) and expected to confirm their neural representations in brain activity in the vmPFC, the VS and the PCC (McClure, 2004; Kable and Glimcher, 2007; Figner *et al.*, 2010). We estimated moral subjective values with a structurally isomorphic computational model; this allowed us to test whether moral subjective values would only be represented by similar structures as financial values (e.g. the vmPFC, Shenhav and Greene, 2010) or whether they instead engage representations in other brain areas [e.g. in the right temporo-parietal junction (rTPJ; Young *et al.*, 2007; Kliemann *et al.*, 2008)], thereby identifying a novel component of the network of brain regions involved in value computations dedicated to representing moral preferences.

It has to be noted, however, that while our task allows us to explicitly compare the neural representation of moral preferences (related to decisions about human lives) and of financial preferences (related to decisions about payoffs at different timepoints), the present task does not allow us to pinpoint exactly which specific differences between the two types of choices may lead to differences in valuation processes. Various candidates for such differences exist in theory (e.g. social *vs* non-social context, medical *vs* financial domain and implicit *vs* explicit numeric information), even though many of them have been shown by other studies to lead to comparable activation of the common currency network (see for instance: Lebreton *et al.*, 2009; Mobbs *et al.*, 2009; Zaki *et al.*, 2011; Camerer and Mobbs, 2017). Irrespective of such considerations, our main aim here was to test whether the moral preferences underlying the present choices rely on neural activity in the common-currency network rather than what precise features of the moral task are mapped onto these preferences. We thus chose a similar strategy as many other studies in the field (see Ruff and Fehr, 2014 for an overview) and contrasted two types of decision-making that differed maximally in the need to draw on moral preferences (e.g. moral *vs* financial) but minimally on visual input, required computations and motor output. If our results identify neural networks specifically involved in computing subjective values for the moral task (relative to the matched financial task), then we can indirectly compare these moral-specific neural activations to those identified in previous studies investigating some of the task dimensions mentioned above (e.g. (Mobbs *et al.*, 2009; Telzer *et al.*, 2013).

## Materials and methods

### Participants

The participants were 25 healthy students from the University of Zurich (age: min 19, max 34, mean = 22.08, SEM = 0.74 years old; 13 females) with no reported history of neurological or psychiatric disorders and no current use of medication as measured with standard surveys. All the experimental procedures were approved by the Research Ethics Committee of the Canton of Zurich.

### Experimental procedures

**Fig. 1.**
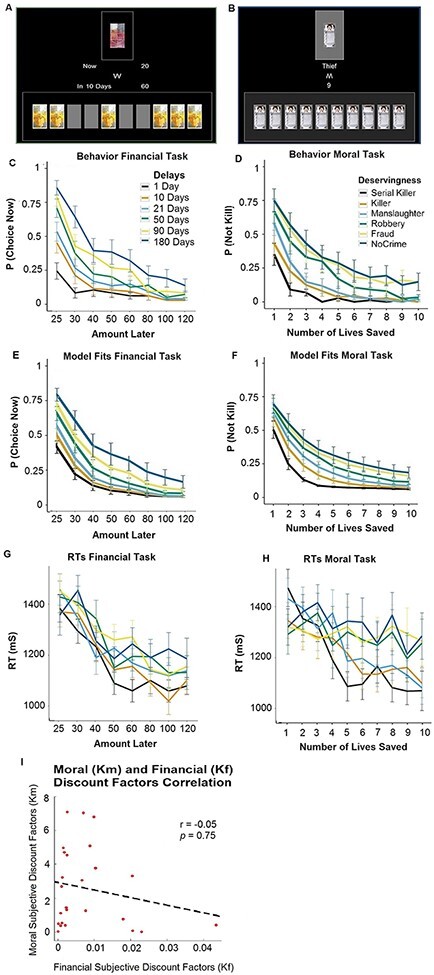
Paradigm and behavioral results: participants made financial (A) and moral (B) choices. In the financial task, they decided whether or not to give up a sooner smaller financial reward for a later larger financial reward. In the moral task, they decided whether or not to sacrifice one coma patient to save a larger group of people requiring organ transplants. (C) The probability of giving up the sooner smaller reward increased as the amount of the delayed reward increased. The increase was modulated by the delay participants had to wait to receive the larger option. (D) Analogously, the probability of killing the one person in order to save the larger group of people increased with the number of people that could be saved; in this case, the probability of choosing to sacrifice the coma patient was modulated by deservingness. Behavior in both tasks was well captured by the models used, as revealed by the model fits for the financial (E) and the moral (F) tasks. Our analyses focused on trials matched for choice (un)certainty, as illustrated by comparable RTs and choice probabilities across the two tasks (G and H; analyses of all trials are reported in SI). We found no evidence of correlation between financial and moral discounting (I).

For both types of decisions, participants selected between two choice alternatives on each trial (see the section Materials and Methods in the supporting information for more information on the tasks): for financial decisions (Figure 1A), participants chose between 20 Swiss Francs (CHF) to be received today or an equal or larger financial reward (min = 20 CHF, max = 120 CHF) paid out after one of six different time delays (min = 1 day, max = 180 days). For moral decisions (Figure 1B), participants chose between saving the lives of a larger number of people (min = 1, max = 10) at the expense of sacrificing the life of one person and not harming the one person and letting the group die. Participants were explicitly informed that the group of people had no prior criminal records. Moreover, closely mirroring the financial task, participants had to consider an associated feature that may discount the choice option´s value: the moral deservingness of the lives at stake, a property known to play an important role in modulating moral decisions (Kliemann *et al.*, 2008; Awad *et al.*, 2018). We implemented this by assigning one of six different prior criminal records (ranging from no criminal record to serial killer) to the single person that could be saved or harmed for the benefit of the group. To ensure that participants performed both tasks based on similar subjectively estimated numerical representations, at the end of the study, participants were asked to self-report their subjective perception of delay and of moral deservingness. These subjective estimates were then entered into the choice models (see below). Importantly, while these models mainly summarize patterns of choices rather than specifying the full set of psychological processes involved in the two tasks, exactly this modeling approach has been used repeatedly and successfully in neuroimaging studies to identify neural subjective value representations in financial temporal discounting tasks (Kable and Glimcher, 2007). Moreover, recent stimulation studies in monkeys have suggested that such neural value representations computed based on models fitted to overt choices can be causally related to choice outcomes (Padoa-Schioppa and Conen, 2017). Thus, the subjective values proposed by these models can be used to identify neural preference representations that are systematically linked to choice outcomes. In other words, relying on these models, we can test if isomorphic algorithms can capture different behavioral and neural implementations. We are not testing whether different models would result in isomorphic neural representations (Lockwood *et al.*, 2020).

Note that while the two tasks are structurally similar, it is of course possible that different psychological processes may be involved in each of the two tasks. Thus, our setup is not ideal for conducting categorical comparisons across different domains (i.e. if one wanted to test if different brain regions are engaged when people take moral *vs* financial choices). However, our analyses did not focus on such categorical comparisons but instead investigated correlations of BOLD signals with either moral or financial *subjective values* that varied substantially from one trial to the next within each of the tasks. All these trial-by-trial correlations with subjective values and comparisons of these correlations between the two tasks, therefore, keep constant any factors that differ between moral *vs* financial decisions *per se*. Thus, our comparisons of SV representations between the two types of choices cannot be confounded by categorical differences between the psychological processes triggered by the two choice contexts.

### Behavioral results

Subjective financial and moral values were estimated based on the participants’ financial or moral choices, respectively. In the reward domain, previous studies have repeatedly shown (Green and Myerson, 2004; McClure, 2004) that discount rates are typically well captured by hyperbolic functions, both in humans and other animals (Frederick *et al.*, 2002; Green *et al.*, 2004). In order to estimate participants’ subjective financial values for each trial, we modeled the behavioral data with a standard hyperbolic function:

(1) $${\textrm{SV\_LL = LL/(1 }} + {K_f}*T)$$

where SV_LL is the subjective financial value of the delayed option estimated as a fraction of the immediate reward, LL is the larger later amount offered, *K_f_* corresponds to a subject-specific financial discounting constant and *T* represents the individually estimated perception [self-reported for each delay by each participant on a scale from 0 (extremely short) to 100 (extremely long)] of the temporal distance for the time people had to wait to receive the reward. Consistent with previous findings (Kable and Glimcher, 2007), our participants’ discounting curves were well captured by this function (Figure 1E; *R*^2^ = 0.98 ± 0.015). Moreover, the financial discount factors (*K_f_*), and hence the SV_LL, varied substantially across participants (ranging from *K_f_* = 3.78 × 10^−5^ to *K_f_* = 0.043; Figure 1I).

Behavior in the moral task was modeled with a structurally equivalent model to the one used in the financial domain. This allowed us to compare the estimated SV for each task both at the level of behavior (e.g. testing for a correlation between the two SV types) and brain activity. Specifically, behavior in the moral task was modeled with the following hyperbolic function:

(2) $${\textrm{SV\_HL}}\;{\textrm{ = HL}}/(1{\ } + {K_m}*D)$$

where SV_HL is the trial-wise subjective moral value of saving the lives of the larger group by sacrificing the life of one person; HL reflects the number of human lives one can save in the larger group; *K_m_* corresponds to a subject-specific moral discount factor and *D* represents the individually estimated perception [self-reported for each deservingness by each participant on a scale from 0 (not at all) to 100 (extremely wrong)] of the crime committed by the person one could sacrifice. Importantly, the logistic model used in our study allows us to test whether subjective moral values are systematically discounted by deservingness: if individuals did not systematically discount the value of human lives, we should observe that a participant essentially chooses to never/always sacrifice a human life to save a larger number following a deontological/utilitarian moral principle. Furthermore, we can also test if a participant’s moral decisions follow a hybrid decision rule, whereby she decides to always sacrifice the life of a person if a given minimum number of lives would be saved, and never to sacrifice this life if less than the given minimum number would be saved.

As a first important result, we found that individual discount curves for moral choices (Figure 1D) were well fit using equation (2) (*R*^2^ = 0.96 ± 0.03; Figure 1F). This finding suggests that the moral subjective values estimated here indeed play an important role in moral decision-making. Furthermore, like in the financial domain, moral subjective values and the moral discounting factors (*K_m_*) varied substantially across participants (ranging from *K_m_* = 9.3 × 10^−2^ to *K_m_* = 7.08; Figure 1I). We found no evidence that subjective moral values are not systematically discounted by deservingness, therefore demonstrating that participants did not follow a simple pre-determined (hybrid) decision rule (always save/kill if above/below a specific number) that would not require engaging in the valuation of moral choice options.

While the two types of choices were comparable in terms of their computational requirements, they obviously differed qualitatively in terms of choice options and their consequences: on the one hand, participants made decisions about whether or not to harm a human to save other lives, while on the other, they decided between different financial payoffs. It may, therefore, be expected that the two types of choices may differ in terms of response difficulty. While the average response times (RTs) for the two types of decisions—a standard proxy to measure task difficulty—were similar [average RTs moral 1214 ms ± 28 (SEM), financial 1235 ms ± 24 (SEM), paired *t*-test, *t*(24) = 0.39, *P* = 0.7], an inspection of the behavioral results (Supplementary Figure S1C and D) revealed a difference in the probability distributions of choosing one of the two options across the two tasks. This difference could indicate that the two tasks may not be fully matched with respect to how subjective values relate to choice (un)certainty (Shenhav *et al.*, 2014, 2016). To control for this potential confound, we focused our SV analyses only on trials that were matched across the two tasks with respect to both RTs and the probability of choosing one of the two options. To achieve this, we excluded from the financial task the two trial types that yielded the highest levels of choice certainty (all trials that offered as a larger later reward 20 or 22 CHF). This exclusion resulted in fully matched choice frequencies and reaction times across both conditions (Figure 1C–D and G–H). We further confirmed this matching in two-sided *t*-tests comparing the standardized slopes (β1) of (i) the logistic regression estimating the probability of choice [see equations (3) and (4) in the behavioral analysis section below] and (ii) a linear regression (formally, RTs = β0 + β1 SV + E) estimating the relation between RTs and moral and financial subjective values for each individual. The *t*-tests revealed no significant difference across the two tasks, neither with respect to choice probability [*t*(24) = 0.29, *P* = 0.77] nor with respect to RTs [t(24) = 0.83, *P* = 0.41]. Thus, following this procedure, the two types of decisions did not differ in terms of choice difficulty, which allowed us to use the model-derived SVs for an unbiased comparison of the underlying neural mechanisms (note that we find similar results when using the complete data-set for this purpose; see supporting information Supplementary Figures S1–S3 and Supplementary Tables S1 and S2).

Interestingly, although financial and moral choices were on average well fitted by identical functions and did not differ with respect to task difficulty/RTs, we could not find behavioral evidence suggesting that moral and financial valuation processes rely on correlated psychological mechanisms: when testing for a relationship between each individual’s discounting in the financial and moral domain, we found no correlation between both discounting factors *K_m_* and *K_f_* (*r* = −0.05, *P* = 0.75, Spearman regression; Figure 1I). This absence of a correlation already suggests that moral and financial value estimations may be performed by independent neural and cognitive decision mechanisms.

### Functional imaging results

**Fig. 2.**
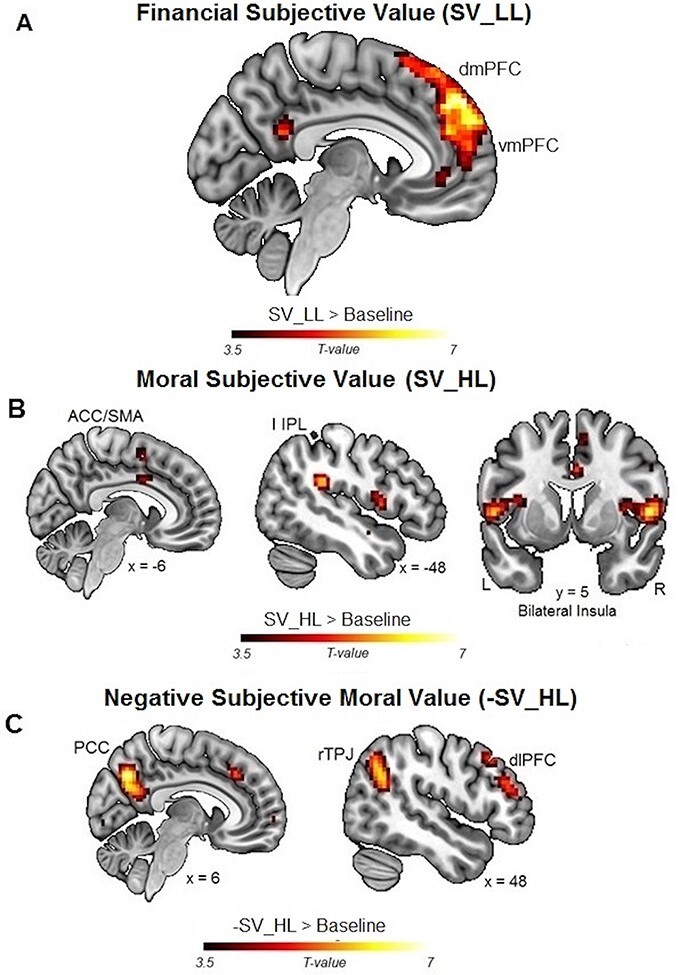
Functional neuroimaging results. (A) Financial subjective values (SV_LL) were represented by neural activity in the mPFC. (B, C) Moral subjective values (SV_HL) were positively represented by neural activity in the bilateral AntIns (B) and negatively (-SV_HL) in the rTPJ, DLPFC and PCC (C).

As an initial imaging analysis step, we confirmed the well-known neural correlates of subjective ‘financial’ values. As expected based on the literature (Kable and Glimcher, 2007; Levy and Glimcher, 2011; Bartra *et al.*, 2013), we found a significant correlation between subjective financial values of the delayed monetary option (SV_LL) and BOLD activity in brain areas associated with subjective financial value processing (Clithero and Rangel, 2014; Grueschow *et al.*, 2015). In particular, we found the hypothesized positive financial subjective value representations in the vmPFC and dmPFC (Figure 2A and Table 1). We did not find any activation reflecting negative subjective financial values (−SV_LL).

**Table 1.** Average brain activity representing subjective moral values positively (SV_HL, rows 4–7, related to Figure 2B) and negatively (-SV_HL, rows 9–12, related to Figure 2C), and average brain activity representing subjective financial values positively (SV_LL, rows 14–18, related to Figure 2A, no activity was found for -SV_LL). All *P*-values are FWE-corrected for the whole brain. SMG = supramarginal gyrus; STS = superior temporal sulcus. Coordinates are listed in montreal neurological institute (MNI) space

Region	Peak-Side	Cluster Size	*x*	*y*	*z*	*Z* score	*T* score	*P*-value
Neural Correlates of Subjective Moral Values (SV_HL)								
ACC		839	0	29	40	5.14	7.06	<0.001
AntIns	R	111	36	2	10	3.73	4.40	<0.001
IPL	L	162	−51	−37	25	4.31	5.38	<0.001
IPL	R	104	60	−16	22	4.61	5.93	<0.001
Negative Neural Correlates of Subjective Moral Values (-SV_HL)								
Cuneus	R	125	12	−85	4	3.92	4.70	<0.001
DLPFC	R	829	54	35	22	4.80	6.30	<0.001
PCC		506	0	−67	37	5.21	7.22	<0.001
TPJ	R	538	48	−58	31	4.55	5.82	<0.001
Neural Correlates of Subjective Financial Values (SV_LL)								
mPFC	L	938	−12	50	49	5.04	6.82	<0.001
SMG	R	273	66	−37	−2	4.62	5.95	<0.001
STS	L	337	−48	−61	25	4.15	5.08	<0.001
STS	R	135	60	−61	28	4.28	5.32	<0.001
Visual Cortex	L	194	−30	−52	−14	5.04	6.83	<0.001

Importantly, our fMRI analysis revealed that the trial-by-trial subjective ‘moral’ values were represented by BOLD signals in a different set of brain regions comprising the bilateral temporo-parietal junction (TPJ), the PCC, the right dorsolateral PFC (rDLPFC), the right anterior insula (AntIns), the left inferior parietal lobule (IPL) and the anterior cingulate cortex (ACC) (Figure 2B and C, and Table 1).

These results allowed us to directly relate individual differences in moral preferences to differences in neural activity in these brain regions, effectively providing novel evidence of a neural signature of subjective moral preferences. More specifically, we found that the higher the subjective moral value of the trial-wise varying numbers of human lives at stake (SV_HL), the higher the BOLD activity in the bilateral AntIns, the left IPL, and the ACC (Figure 2B), and the lower the BOLD activity in the rTPJ, the PCC and the rDLPFC (Figure 2B). These results are generally consistent with previous reports of activity in some of these brain areas during moral decisions (Greene *et al.*, 2001, 2004; Kliemann *et al.*, 2008; Hutcherson *et al.*, 2015a), as well as in the representation of expected values in the moral domain (Shenhav and Greene, 2010), but they now unambiguously reflect subjective moral preferences.

Nevertheless, one may wonder whether our results reflect subjective value representations used to guide choices or may rather be a consequence of these (binary) choices themselves. This is because the tendency to sacrifice the one person to save the group increases with the varying moral subjective value (SV_HL; conversely, the tendency to not sacrifice this person increases with −SV_HL). To investigate this, we ran an additional analysis that, instead of focusing on SVs, identified neural activity correlating with the choice reported by the participants (i.e. moral task: choices not to harm the one person *vs* choices to save the larger groups or vice versa; financial task: choices to keep the smaller immediate reward *vs* the larger later reward or vice versa). These analyses did not reveal significant activations, suggesting that the neural signals identified by the previous analyses indeed reflect subjective value computations and not choice outcomes *per se*.

**Fig. 3.**
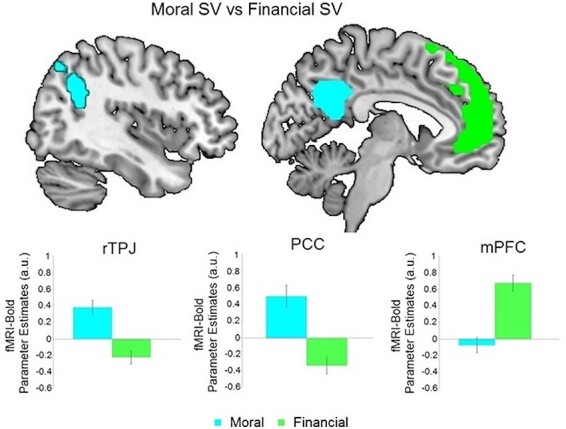
Domain-specific subjective value representations in the financial task: specific neural representations of moral subjective value (-SV_HL) were found in the rTPJ, rDPLFC and PCC (cyan). In contrast, specific correlates of financial subjective value (SV_LL) were identified in the mPFC (green). Colored areas represent clusters of activity specific for each of the two tasks, and not an ROI analysis. These analyses were performed on trials matched on (un)certainty across the two tasks.

A crucial aim of our fMRI analysis was to establish if moral subjective value computations rely on domain-general mechanisms also shared with non-moral value-based decisions (Shenhav and Greene, 2010) or whether they rely on markedly different brain regions. We thus directly compared the neural activity related to SV_HL computations *vs* the activity related to the matched SV_LL computations. This confirmed that the activity in the rTPJ, the rDLPFC and the PCC that correlated negatively with SV_HL (i.e. that coded for the moral value of not harming the one person) was indeed domain specific, as it was significantly stronger than the BOLD correlations with SV_LL (Figure 3 and Table 2). Intriguingly, the activity in the ACC, insula and IPS correlating positively with SV_HL (the moral value of saving the larger group) was not domain specific, i.e. it was not significantly stronger than the BOLD correlations with SV_LL in those regions. In contrast, BOLD activity in the medial PFC (mPFC; Figure 3 and Table 2) was specifically related to representing financial values, since it was significantly stronger than the BOLD activity correlation with moral subjective value (i.e. SV_LL > −SV_HL).

**Table 2.** Average brain activity specifically representing subjective moral values > subjective financial values (rows 4–7), and average brain activity specifically representing subjective financial values > subjective moral values (rows 9–10), related to Figure 3. These analyses were performed on trials matched for (un)certainty across the two tasks. All *P*-values are FWE-corrected for the whole brain. STS = superior temporal sulcus. Coordinates are listed in MNI space

Region	Peak Side	Cluster Size	*x*	*y*	*z*	*Z* score	*T* score	*P*-value
Neural Correlates of Subjective Moral Values (-SV_HL) > Subjective Financial Values (SV_LL)								
Cuneus	R	612	15	−88	7	5.59	8.16	<0.001
DLPFC	R	83	54	29	16	4.78	6.26	<0.001
PCC		455	0	−67	37	5.59	7.31	<0.001
TPJ	R	283	57	−61	28	5.43	7.73	<0.001
Neural Correlates of Subjective Financial Values (SV_LL) > Subjective Moral Values (-SV_HL)								
mPFC	L	1314	−15	26	55	5.54	8.01	<0.001
STS	L	347	−42	−64	37	4.60	5.91	<0.001

We further confirmed that financial subjective values were more strongly represented in regions of the common-currency network by performing regions of interest (ROI) analyses (8 mm sphere centered at peak coordinates; Supplementary Table S3 in the SI). These analyses tested for neural activity in brain regions proposed by previous meta-analysis to represent financial SVs (Bartra *et al.*, 2013). Confirming our whole-brain analysis, this ROI approach also revealed that only financial subjective values were represented within the neural-common-currency network (SV_LL > −SV_HL; *t*-test, all *P*s < 0.001; Supplementary Table S3 in the SI). We also tested for potential functional activity involved in computing both moral and financial subjective values. The conjunction analysis testing for such overlap in coding of both SV_LL and SV_HL, however, did not reveal any significant result. These findings highlight that the neural representation of moral subjective values relies largely on domain-specific mechanisms at least in contexts where moral considerations do not need to be compared to monetary amounts as here. Note that this conclusion is not at odds with findings that the valuation regions like the vmPFC may play a role in moral decision-making when this involves trade-offs between moral and financial values, as in previous studies (Dogan *et al.*, 2016; Crockett *et al.*, 2017). However, our findings demonstrate that the computation and representation of purely moral subjective values seem to involve a distinct set of areas.

## Discussion

We identified neural value representations that underlie individual moral preferences, thereby testing if the brain represents moral and material preferences within a common neural currency neural network. This hypothesis that neural value processes are shared between moral and material preferences appears at odds with the common moral intuition that human lives should not be valued in material terms. To clarify this discrepancy, we employed a novel moral choice paradigm allowing us to investigate how the human brain represents the subjective value of saving/harming the life of other persons and to compare these neural processes with those representing financial subjective value. Our behavioral models show that both financial and moral decisions concerning who should be saved/harmed are similarly well fit by structurally isomorphic computational decision models. Neurally, we found that moral subjective values are computed according to similar principles as financial values but are represented in domain-specific brain areas that differ markedly from those involved in financial valuation. Importantly, in the present paper, we can only demonstrate that these neural networks represent subjective moral values more than financial values. Specifically, we identify neural processes involved in assigning value to human lives that are clearly dissociable from the neural processes assigning value to money. Our data do not, however, allow us to estimate the degree to which the neural networks representing these moral preferences may be involved in choices based on other types of moral or social preferences. We therefore only claim that the moral preferences studied here recruit activity in this network rather than in the common currency network but not that the identified neural regions are functionally specific to all possible types of moral valuation. Future studies should investigate this specificity further by directly comparing the neural representations of moral preferences to those of social and other preferences. Thus, when we refer to ‘moral-specific’ networks in this paper, we always refer to the relative specificity of moral preferences for human lives *vs* financial preferences.

More specifically, our data show that subjective moral values (±SV_HL) are represented in a network of regions comprising the rTPJ, the PCC, the rDLPFC, the left IPL and the AntIns (Figure 3 and Table 2). Importantly, directly comparing the neural activity elicited by moral *vs* financial subjective values allowed us to demonstrate that purely moral subjective values are not represented within the set of regions comprising the network found representing the value of material types of goods (Izuma *et al.*, 2008; Zink *et al.*, 2008; Levy and Glimcher, 2012). This suggests that the brain comprises a valuation system beyond the traditional common-currency network that is more sensitive to moral values than monetary values. The areas in this moral valuation network have previously been shown to respond to socially salient components of decisions, such as other’s pain (Decety and Lamm, 2007; Santiesteban *et al.*, 2012) or potential harm (Crockett *et al.*, 2017). Importantly, we identified these areas through a novel paradigm that allowed us to study moral and financial values independently, instead of studying these values in a context where both need to be integrated and/or compared. While previous research has suggested that the final overall value of choice options combining both moral and financial considerations may be represented in vmPFC activity (Crockett *et al.*, 2017), our results demonstrate that moral values *per se* are computed in different areas before being available for comparison and integration with material values. Future studies should examine the processing steps in this moral value network and the precise mechanisms by which different types of values are ultimately integrated in the vmPFC and interconnected areas.

We found that neural moral value representations of harming one specific person (captured in our model by −SV_HL) were specifically expressed for moral but not financial choice problems; this was not the case for the neural activity correlating positively with the number of lives in the larger group one could save (captured in our model by SV_HL). Thus, our findings support the view that subjective moral valuations recruit both moral-specific valuation mechanisms and domain-general decision processes as previously identified (Shenhav and Greene, 2010). This previous study found that certain choice-relevant information about the options in moral decisions (such as magnitudes and probabilities of outcomes) are represented by domain-general neural mechanisms. In particular, it was found that computations of the expected value of probabilistic outcomes in moral scenarios elicited neural activity in regions commonly associated with computations of the expected value of probabilistic financial rewards, such as the striatum (Tobler *et al.*, 2006) and the vmPFC (Knutson and Peterson, 2005; Hare *et al.*, 2008). However, as this study focused on representation and computation of objective information (such as probability, magnitude and expected values), it does not inform us with respect to how the brain represents and derives subjective moral preferences that differ between individuals with different moral stance. Nevertheless, some aspects of our results are consistent with this previous study since we found that BOLD correlates of the moral value computations taking into account the numbers of lives that one could save in the larger group (i.e. SV_HL) did not differ from those involved in financial choices relying on similar magnitude estimations. Thus, our results show that from the perspective of neural coding, the moral values used for choices comprise both a subjective, domain-specific component and a domain-general component shared with financial choices. Our results do not reveal, however, how these two types of value representations are integrated by the brain when a trade-off between moral and financial values is required. However, previous studies have investigated decisions in contexts that require the direct integration of moral and financial values (e.g. deciding between donating to a charity or keeping the money for oneself). These studies found that the combined subjective value of choice options was mostly represented in the vmPFC but modulated by social information from the TPJ (Hare *et al.*, 2010; Crockett *et al.*, 2014; Strombach *et al.*, 2015; Soutschek *et al.*, 2016). In this decision context, the rTPJ has been thought to estimate socially salient components, such as the need to overcome one’s perspective or the deservingness of a charity, and to pass this information on to the vmPFC where the value computation is ultimately implemented (Hare *et al.*, 2010; Strombach *et al.*, 2015). However, in all these studies, the choice options resulted in financial payoffs, meaning that these studies cannot determine whether the vmPFC activity represented financial values (that were modulated by moral concerns) or the moral values themselves. Thus, our results offer a novel and intriguing perspective on the role of the rTPJ in moral value computations. For decisions based only on moral values (i.e. where there is no trade-off between self-interested financial values and moral values), our data suggest that subjective moral values can be represented directly in the rTPJ without any vmPFC involvement. This suggests that moral preferences originate from the idiosyncratic structural and functional properties of rTPJ (and the other areas we identified) rather than from value coding in the vmPFC. The fact that we did not find that this region was involved in moral value computations does not exclude the possibility that moral value may be represented in other aspects of neural activity that our analysis methods were not designed to detect. For example, future studies employing multivariate analyses may clarify whether more fine-grained neural activity patterns (rather than broad univariate signal changes) in vmPFC may correlate with the subjective moral values in our paradigm.

Furthermore, comparing the behavioral and neural responses to the material and the moral value-estimation contexts, one additional intriguing asymmetry emerges: while the neural responses to the financial subjective value only manifests in one direction (i.e. positive correlation formally captured by SV_LL), the neural responses to the moral ‘subjective value’ reveal two distinct patterns, namely two distinct sets of brain areas are sensitive to the increase or decrease of the moral subjective value of harming one person (which are formally captured as SV_HL and −SV_HL in our models). These distinct neural networks could reflect different types of moral considerations required for constructing moral values, with the neural correlates of SV_HL representing non-consequentialist moral preferences and −SV_HL capturing utilitarian-like moral preferences. It is therefore conceivable that the subjective moral values informing the participants’ behavioral response results from the integration of signals from both of these neural networks. Interestingly, this pattern was only observed in the moral context but did not emerge in the financial context. However, within this comparison of moral *vs* financial value, it is legitimate to infer that the differences in the neural correlates of the two types of value signals cannot be reduced to qualitative differences across the two tasks. Moral preferences (formalized in SV_HL and −SV_HL) and financial preferences (SV_LL) correlate with activity in different neural networks in a within-task and between-subject comparison. Such differences cannot reflect the categorical task set *per se* but need to relate to individual and between-trial differences in those psychological processes that assign value to the specific choice options present on each trial—which may involve empathy, perspective taking or arousal for the moral task or reward processing, emotion and cognitive control for the financial task. As noted above, it is, of course, possible that our two choice tasks differ with respect to the psychological processes involved in the two types of decision contexts. For instance, the two tasks could differ with respect to the amount of imagination/perspective taking involved to make a choice.

However, note that any such possible differences are very unlikely to have confounded our results. First, these differences would mainly have affected categorical differences (i.e. comparisons of all moral *vs* all financial trials), which we did not test for here. Instead, we tested for differences in correlations of BOLD with moral SVs *vs* with financial SVs. These SVs varied across trials and individuals, so any constant differences between the two types of trials was controlled for in our analyses (it is highly implausible that imagination/perspective-taking should correlate systematically with SVs in just one domain but not the other). Second, it is actually unclear if and to which extent financial and moral decisions differ along these dimensions. Based on previous studies of hypothetical *vs* real decisions across different domains (Feldman Hall *et al.*, 2012; Camerer and Mobbs, 2017), one would expect the imagination network to comprise the PCC but also the mPFC and the posterior hippocampus. However, we found that one of these regions (the mPFC) specifically represented financial subjective values, whereas another of these regions (the PCC) specifically represented moral subjective values. Moreover, one previous study (Soutschek *et al.*, 2016) demonstrated a causal involvement of perspective-taking processes in the TPJ in classic financial intertemporal choices. Thus, our results are very unlikely to be influenced by general differences in psychological processes triggered by the different choice contexts; they are much more likely to reflect specifically the moral and financial subjective values that varied across trials and individuals. However, to establish that the identified neural networks are specifically related to the moral elements of decisions rather than other factors, future studies may use variants of the design we establish here to directly examine all the potential differences between the current moral *vs* financial comparisons (e.g. social *vs* non-social settings, self- *vs* other-related choices and hypothetical *vs* real choices). Importantly, previous studies have already established that differences along many of the theoretically possible task dimensions are unlikely to account for our present findings. For instance, previous research has demonstrated that both non-social delay and social discounting are correlated at the behavioral level (Rachlin and Jones, 2008; Rachlin and Jones, 2008) and are both represented within the common-currency neural network (Hill *et al.*, 2017), as well as by networks sensitive to socially relevant cognitive functions [such as theory of mind (Soutschek *et al.*, 2016]. These common neural effects may reflect that the social and temporal discounting tasks both entail financial payoffs (that are discounted by either moral or temporal considerations), as is also the case for other studies that investigated how moral considerations change the valuation of money (Crockett *et al.*, 2017). However, there is also ample evidence that non-monetary social values (e.g. triggered by positive social feedback) are represented in the common-currency network (see for instance: Lebreton *et al.*, 2009; Mobbs *et al.*, 2009; Zaki *et al.*, 2011; Camerer and Mobbs, 2017). Furthermore, previous studies found that values related to both self- and other-oriented outcomes are represented by the common-currency neural network (Telzer *et al.*, 2013), and similar results were found for the comparison of hypothetical vs. real rewards (Kang *et al.*, 2009, 2011; Camerer and Mobbs, 2017). Thus, it seems implausible that the moral-specific value representations we identified here reflect general differences between choices that are social *vs* non-social and self-referential *vs* other-referential or associated with hypothetical *vs* real consequences. Nevertheless, the specific features of our moral task giving rise to the distinct neural value representations should be confirmed by future studies directly comparing the neural correlates of these features.

Relating moral preferences to neural activity, we found that SV_HL was negatively associated with neural activity in the rTPJ, PCC and DLPFC among other areas (Table 1 and Figure 2C) and positively associated with activity in the AntIns and the left IPL (Table 1 and Figure 2B). These findings suggest a mechanistic interpretation of how moral preferences in our choice context are represented in the brain. That is, SV_HL may be computed based on assessments of the harm inflicted on the one person who may be killed as a consequence of one’s choice, consistent with previous studies linking brain activity in the rTPJ, PCC and DLPFC to processing harm aversion and empathy (Crockett *et al.*, 2010, 2017; Majdandžić *et al.*, 2012; Ugazio *et al.*, 2014). Thus, this neural activity could be interpreted as encoding the increase in the value of a human life related to its increasing moral deservingness (which correlates negatively with SV_HL and positively with TPJ/PCC/DLPFC activity). Conversely, the moral preference that may consider it required to save a larger number of people could rely on neural valuation mechanisms responsible for comparing the magnitudes of the moral choice options, reflected in brain activity in the left IPL and in the AntIns that was not truly domain-specific (i.e. not significantly stronger than the corresponding correlations with the magnitudes of financial values). The left IPL has been associated with magnitude representations and reasoning processes (Goel *et al.*, 2017), while the AntIns has been associated with representing social arousal and emotions elicited by socially salient stimuli (Lindquist *et al.*, 2015). An increase of AntIns activation, in this case, could thus reflect the increased arousal resulting from the increased evidence endorsing a harming action [killing the patient to save a large number of lives; see also (Poppa and Bechara, 2015)]. This interpretation accommodates and extends the ideas proposed in the previous study that identified a positive correlation between the activity in these brain areas and an increase of expected value in moral decisions with probabilistic outcomes (Shenhav and Greene, 2010). Taken together, our results thus suggest that moral preferences are encoded by (at least) two antagonistic neural systems rather than by a unitary neural network as is the case for financial preferences.

The analysis of the monetary control task showed that financial subjective values were indeed represented by neural activity in the vmPFC and PCC, consistent with numerous previous findings (Kable and Glimcher, 2007). These value-computation mechanisms may well be domain general to some degree and contribute to moral decisions that require the representation and integration of objective information about choice outcomes [such as the magnitude and probability of achieving an outcome (Shenhav and Greene, 2010)] or its ‘utility’ to the agent (Hutcherson *et al.*, 2015b). Such domain-general mechanisms for information representation can even be useful to anticipate individual sensitivity to different features of the choice options: for instance, it was shown (Shenhav and Greene, 2010) that participants’ sensitivity to the probability of outcomes had higher AntIns activity or that participants who were more sensitive to magnitudes displayed higher vmPFC and IPL activity. However, since this study did not identify moral preferences in terms of subjective moral values, it is currently unclear how these preference computations may relate to, or be influenced by, individual differences in representations of choice options and their characteristics. Nevertheless, these domain-general mechanisms seem to have an important role for moral values when these are traded off with other types of values, for instance, in situations where financial valuation mechanisms corrupt human moral values (Falk and Szech, 2013) or where moral values related to the aversion of harming others can discount financial values (Crockett *et al.*, 2014). This raises the interesting question for future studies what context factors may determine how domain-general valuation mechanisms compete and interact with the moral-specific mechanisms identified here and which higher-level areas may control the interaction of the different valuation systems.

More generally, while trolley-type moral dilemmas have been questioned for their ecological validity (Kahane, 2015), recent technological developments in robotics and artificial intelligence have revitalized the importance of studying human decision-making in these type of dilemmas (Bonnefon *et al.*, 2016). Moreover, a previous cross-cultural study (Awad *et al.*, 2018) demonstrated that while there seem to be general, culture-independent moral preferences (such as saving a larger number of lives), the situational factors influencing moral preferences strongly vary across cultures and countries. In our study, we show that differences among moral preferences can already be detected at the individual level and can be explained using a simple subjective-value computational model. Consistent with the previous behavioral study (Awad *et al.*, 2018), we found a general preference for saving larger number of lives for all our participants. However, this general preference strongly interacted with each participant’s subjective perception of moral deservingness of the lives involved, and both these factors jointly determined the resulting subjective moral values. Thus, our results provide critical information on the origins of individual and cultural differences in moral preferences and may be important for future ethical, public and scientific debates regarding decisions taken by artificial intelligence. For instance, during the SARS COVID-19 pandemic, it has been frequently debated how intensive-care-unit beds should be allocated to patients in case a given hospital runs out of space: should those with highest chances of surviving always be prioritized or should we use some other criteria (Ballantyne *et al.*, 2020)? Our current results identify distinct neural mechanisms by which our brains compute trade-offs between saving and harming human lives, which differ from neural valuation processes involved in selecting between material goods. This suggests that artificial intelligence may benefit from accounting for the properties of these mechanisms in order to be perceived as morally appropriate. Last but not least, our study illustrates how moral preferences may be assessed in a manner that is computationally similar to the assessment of financial preferences without requiring the participants to read and understand complex moral vignettes. This facilitates the identification of the choice-related brain mechanisms and may prove essential for moving toward an integrated perspective of how the brain controls and integrates moral and material concerns in the control of actions, in particular, in situations where these two types of concern may compete or interact (e.g. in philanthropy or sustainable finance).

## Supplementary Material

nsab100_Supp
